# Cochleovestibular Signs As the First Manifestation of Fabry Disease: A Case Report and Literature Review

**DOI:** 10.7759/cureus.57289

**Published:** 2024-03-30

**Authors:** Yasmina Zakaria, Naji Yahya, Najib Kissani

**Affiliations:** 1 Neurology, Mohamed VI University Hospital of Marrakesh, Cadi Ayad University, Marrakesh, MAR; 2 Neuroscience, Neuroscience Research Laboratory, Marrakesh Medical School, Marrakesh, MAR; 3 Neurology, Agadir University Hospital, Agadir, MAR; 4 Research, Neurosciences Innovation Cognition Ethique (NICE) Research Team, Rein Endocrinologie Gastroentérologie Neurosciences Ethique (REGNE) Research Laboratory, Faculty of Medicine and Pharmacy, Ibn Zohr University, Marrakesh, MAR

**Keywords:** fabry disease, angiokeratomas, acroparesthesia, tinnitus, hearing loss, fabry's disease

## Abstract

Fabry disease (FD) is an X-linked lysosomal storage disorder characterized by alpha-galactosidase A deficiency, resulting in globotriaosylceramide accumulation and diverse clinical manifestations. We report a case of a 22-year-old male presenting with cochleovestibular disorders as the initial FD manifestation, alongside a literature review. Diagnostic evaluation revealed reduced alpha-galactosidase A activity, confirming FD. Cochleovestibular involvement, although underexplored, significantly affects FD patients, often presenting with sudden deafness or sensorineural hearing loss. Prompt diagnosis and enzyme replacement therapy are crucial for managing FD. Otolaryngologists play a key role in early detection and intervention. This case underscores the importance of considering FD in cases of hearing loss, tinnitus, or vertigo, emphasizing the need for heightened awareness among healthcare providers.

## Introduction

Fabry disease (FD) is an X-linked lysosomal storage disorder caused by a deficiency in alpha-galactosidase A, leading to the accumulation of globotriaosylceramide in plasma and lysosomes. This aberration leads to variable clinical complications [1,2], ranging from stroke/ transient ischemic attack to cardiac manifestations and renal failure [3]. The otologic implications of FD have been reported over the past 15 years, marked by progressive sensorineural hearing loss, particularly at high frequencies, and a higher incidence of sudden deafness compared to the general population [4,5]. Vestibular involvement, however, has been relatively understudied, with reported prevalence rates ranging from 25% to 80% [6]. A specific enzyme treatment has been developed over the last ten years, slowing the progression of the disease [7]. In this study, we report a case of FD presenting with cochleovestibular disorders as the first manifestation, which is an uncommon onset presentation, with a review of the literature.

## Case presentation

We report the case of a 22-year-old patient, without any past medical history, admitted to our neurology department for a three-month history of left-sided hearing associated with right-sided permanent tinnitus and intermittent vertiginous sensations lasting from a few hours to days, the whole evolving in the context of apyrexia and preservation of general condition. On examination, the patient was hemodynamically and respiratorily stable. Neurological examination revealed normal muscle strength and normal deep tendon reflexes in the four limbs. No truncal ataxia with no slow or hypermetric saccades. A hypersensitivity to thermal and pain stimulation with acroparaesthesia was found. The cognitive assessment showed mild deficits in attention, with no abnormalities in the cognitive evaluation. The otoscopy examination was normal, while the vestibular assessment revealed rotatory nystagmus, with a negative Fukuda test and a head-shaking test. The Dix and Hallpike maneuvers were unremarkable. overall examination found angiokeratomas in both thighs.

**Figure 1:**
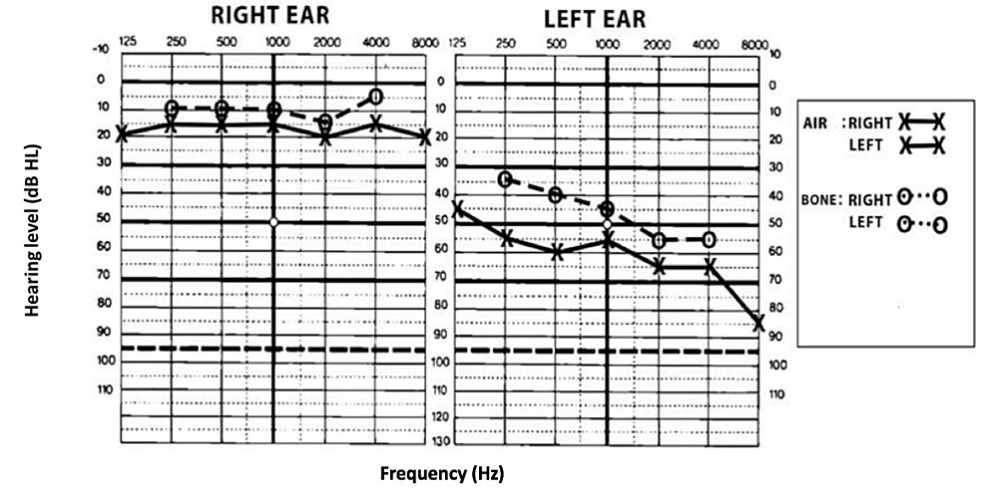
Results of pure tone audiometry show mixed hearing loss in the left ear with an air-bone gap of 15 dB

The tympanogram and otoacoustic emissions were normal. Pure tone audiometry revealed a mixed hearing loss in the left ear, with an air-bone gap of 15 dB on three successive audiograms spaced for 15 days (Figure 1).

A videonystagmography was performed and revealed left areflexia. Saccades were absent. The video head impulse test showed overt saccades. The electroneuromyography study was normal. Laboratory evaluation of the complete blood cell count found normochromic normocytic anemia (Hb: 10.4 g/dl, VGM: 90 fl, TCMH: 30 g/100 ml), biochemical tests (sodium, potassium, phosphocalcic, and glucose), serum protein electrophoresis, blood hepatitis B and C, HIV serology, antinuclear antibody titer, and thyroid tests were normal. Blood urea nitrogen and creatine were above the normal rate (urea: 0.9 g/L, creatinine: 36 mg/L). Based on the clinical presentation of a young male with acroparesthesia, angiokeratomas, hearing impairment, renal dysfunction, and anemia, FD was suspected, and the alpha-galactosidase enzyme assay revealed levels below 1%. The patient underwent enzyme replacement therapy with agalsidase beta, administered via intravenous injections every two weeks at a dose of 1 mg/kg. Additionally, the patient received a conventional left-ear hearing aid and completed 20 sessions of vestibular rehabilitation. The course of treatment resulted in a partial improvement in cochleovestibular disorders leading to complete resolution of vertigo and improvement in hearing loss and tinnitus, accompanied by a reduction in painful episodes of acroparesthesia and stabilization of renal function in five years of follow-up.

## Discussion

FD is a rare X-linked genetic disorder, typically occurring in one in 117,000 births, although recent Italian research suggests a higher incidence, with one in 3,100 male newborns showing alpha-galactosidase A deficiency. Its nonspecific clinical features likely lead to underestimation [8,9]. The Fabry registry, comprising 1,765 patients, reveals an equal sex ratio, yet hemizygous males experience earlier and more severe symptoms compared to heterozygous females [10]. Although geographic variations exist, no ethnic predisposition has been identified. Cochleovestibular involvement affects roughly 80% of patients, with symptoms ranging from sudden deafness to progressive sensorineural hearing loss [3]. The precise histopathogenesis remains inadequately understood, although evidence suggests a reduction in the number of outer hair cells and atrophy of the stria vascularis. Accumulation of glycosphingolipids has been observed in endothelial cells and various ganglia in certain patients. Some researchers have observed cellular depletion within the vestibular ganglion [11]. Further histological studies are needed to fully understand the disease's effect on the inner ear. Diagnosing FD is typically straightforward in males, but it poses challenges in females and individuals with genetic variations [12]. A thorough diagnostic approach, including detailed medical and family history, physical examination, clinical and biochemical assessments, genetic testing, various imaging techniques, and expert consultation, is recommended [13-14]. Specific clinical findings, such as acroparesthesia, angiokeratomas, and cornea verticillate, may provide valuable diagnostic clues [15]. In males suspected of FD, measuring alpha-galactosidase A activity is pivotal, with levels below 1% strongly indicative of classic FD [16]. However, in females, alpha-galactosidase A activity can fluctuate and may remain within the normal range despite clinical manifestations, yet many still develop significant disease [17,18]. Cochleovestibular involvement typically emerges subsequent to renal, cardiac, neurological, and pulmonary manifestations, commonly after reaching the age of 30 [3,11].

Painful episodes, including acroparesthesia and migraine, notably diminish in frequency. Hearing impairment predominantly manifests as endocochlear sensorineural loss, occasionally presenting as a mixed type, often accompanied by negative otoacoustic emissions. Onset may be sudden, attributed to acute cochlear ischemia, or gradually worsen over time due to chronic cochlear ischemia. Vertigo episodes may originate from either peripheral or central sources, potentially associated with cerebrovascular pathology. Infrequently, episodes of vertigo and tinnitus may manifest during childhood, along with rare occurrences of childhood deafness [19]. Treatment of FD primarily relies on enzyme replacement therapy (ERT), with agalsidase alfa and agalsidase beta being the main options. While agalsidase beta is approved in the US, both forms are used in Europe. Intravenous injections are administered biweekly, with doses varying based on the enzyme type. ERT is recommended for symptomatic men aged 18 and above, symptomatic women, and children with severe manifestations. However, long-term efficacy remains uncertain. Two databases, the "Fabry registry" and the "Fabry outcome survey," have been established to gather clinical data and assess ERT efficacy, facilitating disease management. Additionally, novel therapies such as chaperone therapy are under investigation to augment residual enzymatic activity [20].

## Conclusions

FD is a rare genetic disorder that warrants attention from otolaryngologists. Approximately 80% of patients experience cochleovestibular involvement, presenting as sensorineural hearing loss, sudden deafness, vertigo, or tinnitus. Regular auditory screening is essential for the early detection of hearing impairment in Fabry patients. Early diagnosis is crucial for initiating enzyme replacement therapy promptly, which can mitigate irreversible organ damage and improve the long-term prognosis. Otolaryngologists may be the first to suspect FD in cases of unilateral or bilateral sensorineural hearing loss in children, isolated tinnitus, or recurrent episodes of sudden deafness.
